# Endophytes Alleviate Drought-Derived Oxidative Damage in *Achnatherum inebrians* Plants Through Increasing Antioxidants and Regulating Host Stress Responses

**DOI:** 10.1007/s00248-024-02391-2

**Published:** 2024-05-17

**Authors:** Xiumei Nie, Zhenrui Zhao, Xingxu Zhang, Daniel A. Bastías, Zhibiao Nan, Chunjie Li

**Affiliations:** 1 State Key Laboratory of Herbage Improvement and Grassland Agro-ecosystems, College of Pastoral Agriculture Science and Technology, Lanzhou University, Lanzhou, 730020 China; 2 Grasslands Research Centre, AgResearch Limited, Palmerston North, 4442 New Zealand

**Keywords:** *Epichloë*, Plant stress tolerance, Soil moisture, Oxidative stress, Reactive oxygen species, Plant transcriptomes

## Abstract

Endophytes generally increase antioxidant contents of plants subjected to environmental stresses. However, the mechanisms by which endophytes alter the accumulation of antioxidants in plant tissues are not entirely clear. We hypothesized that, in stress situations, endophytes would simultaneously reduce oxidative damage and increase antioxidant contents of plants and that the accumulation of antioxidants would be a consequence of the endophyte ability to regulate the expression of plant antioxidant genes. We investigated the effects of the fungal endophyte *Epichloë gansuensis* (C.J. Li & Nan) on oxidative damage, antioxidant contents, and expression of representative genes associated with antioxidant pathways in *Achnatherum inebrians* (Hance) Keng plants subjected to low (15%) and high (60%) soil moisture conditions. Gene expression levels were measured using RNA-seq. As expected, the endophyte reduced the oxidative damage by 17.55% and increased the antioxidant contents by 53.14% (on average) in plants subjected to low soil moisture. In line with the accumulation of antioxidants in plant tissues, the endophyte increased the expression of most plant genes associated with the biosynthesis of antioxidants (e.g., *MIOX*, *crtB*, *gpx*) while it reduced the expression of plant genes related to the metabolization of antioxidants (e.g., *GST*, *PRODH*, *ALDH*). Our findings suggest that endophyte ability of increasing antioxidant contents in plants may reduce the oxidative damage caused by stresses and that the fungal regulation of plant antioxidants would partly explain the accumulation of these compounds in plant tissues.

**Supplementary Information:**

The online version contains supplementary material available at 10.1007/s00248-024-02391-2.

## Introduction

Plants inevitably withstand various environmental stresses during their lifetime. Drought has become one of the most important limiting factors in global agricultural production, with losses estimated in around $30 billion in the last decade [1]. Under the current global warming, drought events are predicted to become more frequent and the aridity is more severe in most semi-arid and arid areas [2]. The association of plants with beneficial endophytic fungi generally enhances the plant resilience to harsh environments [3–5]. Poöideae (family Poaceae) grasses that form associations with beneficial and asexual fungal endophytes of the genus *Epichloë* are examples of this [6, 7]. These endophytes promote changes in plant morphology, physiology, and biochemistry that increase the host resistance/tolerance to stresses including drought (e.g., root architecture, stomata regulation, and antioxidant contents) [6, 8, 9].

The perennial bunchgrass *Achnatherum inebrians* (Hance) Keng that is widely distributed in arid and semi-arid regions of northwestern China forms symbiotic relationships with either *Epichloë gansuensis* (C.J. Li & Nan) or *Epichloë inebrians* (C.D. Moon & Schardl) [7, 10, 11]. Compared to sexual endophytes, asexual *Epichloë* endophytes generally form mutualistic relationships with their hosts, asymptomatically colonize plant foliar tissues, and are maternally inherited through mycelia established in mature seeds [12, 13]. *Epichloë* spp. associated with *A. inebrians* plants usually enhance the host tolerance and resistance to stresses [14–16]. In drought situations, these endophytes increase the plant tolerance to the stress through promoting the host seed germination, biomass, nutrient acquisition, and photosynthetic rate [17–19]. Additionally, the *E. gansuensis*–mediated drought tolerance in *A. inebrians* plants is partially attributed to the endophyte’s ability to regulate the expression of plant genes involved in the biosynthesis hormones and cuticular wax [20, 21].

Reactive oxygen species (ROS) are byproducts of aerobic metabolic processes in living organisms and include superoxide anions (O^2−^), hydrogen peroxide (H_2_O_2_) and hydroxyl radicals (OH^−^) [22]. Excessive accumulation of ROS in cells can cause oxidative stress, which damages DNA, lipids, and proteins, disrupting normal metabolism and leading to cell death [23]. Oxidative damage in cells can be determined by quantifying the concentration of malondialdehyde (MDA) compounds that are generated in the ROS reactions with lipids [24]. The accumulation of ROS in cells can be prevented by the presence of enzymatic and non-enzymatic antioxidants. These compounds efficiently scavenge ROS and in stressful conditions substantially minimize the potential damage caused by ROS to plant cells [25]. Enzymatic antioxidants include catalase (CAT), peroxidase (POD), and superoxide dismutase (SOD), while non-enzymatic antioxidants include ascorbic acid (AsA), carotenoid (Car), glutathione (GSH), and proline (Pro) [22, 26].

*Epichloë* endophytes usually increase the contents of antioxidants in plants experiencing environmental stresses [27, 28]*.* In drought stress, *Epichloë* spp. have been shown to enhance the plant contents of distinct enzymatic and non-enzymatic antioxidants including ascorbate peroxidase (APX), CAT, glutathione reductase (GR), and Pro [29, 30]. Similar endophyte-based accumulation of antioxidants has been documented in *A. inebrians* plants exposed to distinct environmental stresses [31, 32]. The accumulation of antioxidants in endophyte-infected plants under stressful conditions may counteract the oxidative damage caused by the stresses and ultimately increase the plant fitness [9, 27]. In fact, the increased antioxidant contents documented in *Epichloë*-infected *Elymus dahuricus* plants under drought stress were correlated with reduced oxidative damage and increased plant biomass [33]. Although not entirely understood, the ability of *Epichloë* to stimulate the accumulation of antioxidants in plant tissues would be associated with the endophyte regulation of the plant production of antioxidants and/or the fungal production of these compounds [34, 35]. For instance, the expression of the plant genes *CsFe-SOD*, *CsMn-SOD*, *CsCAT1*, and *CsPRR7* associated with SOD and CAT pathways in *Citrus sinensis* was increased by the association with distinct beneficial fungi (e.g., *Acaulospora scrobiculata, Piriformospora indica*) [36]. Similarly, the inoculation of *Funneliformis mosseae* mycorrhizal fungi in *Poncirus trifoliata* increased the expression of the plant genes *PtMn-SOD*, *PtCAT1*, and *PtPOD* also associated with SOD and CAT pathways in response to drought stress [37].

Here, we evaluated the oxidative damage and antioxidant responses of plants associated with *Epichloë* endophytes subjected to contrasting soil moisture conditions. Plants of *A. inebrians* infected with (EI) and free (EF) of the endophyte *E. gansuensis* were grown in low and high soil moisture conditions. The levels of oxidative damage, contents of antioxidants, and the expression of plant genes coding for enzymes associated with antioxidant pathways were assessed. We hypothesized that the presence of endophytes in plants would simultaneously reduce the oxidative damage and increase the contents of antioxidants, especially at low soil moisture. We also hypothesized that the endophyte effects on the expression of plant genes associated with antioxidant pathways would be aligned with changes in the patterns of accumulation/reduction of antioxidants across soil moisture conditions.

## Materials and Methods

### Plant Material and Experimental Treatments

A few leaf sheaths of around 100 individual *A*. *inebrians* plants (with 15–30 tillers) were harvested, stained with aniline blue, and examined by microscope (× 40, Leica DMRB, Germany) in 2011. Almost all these plants were infected with the endophyte. Seeds were collected from the EI plants, and a subset of 50 seeds per plant were randomly selected to determine the endophyte infection through microscopy using a similar protocol to that used for leaf sheaths [10]. The endophyte was present in all seeds (50 out of 50 per EI plant). Seeds from EI plants were pooled together and labeled as the F0 seed lot. To obtain seeds free of *E. gansuensis*, half of the seeds from F0 seed lot were treated for 2 h with the fungicide thiophanate-methyl (70%, diluted 100 times) and the other half were not treated with any fungicide [38]. To obtain stocks of EI and EF seeds for experimentation, 200 seedlings from fungicide-treated seeds and 200 seedlings from untreated seeds were sown separately in the experimental field at the College of Pastoral Agricultural Science and Technology located in the Yuzhong Campus of Lanzhou University (Lanzhou, China; 104° 39′ E, 35° 89′ N, altitude 1653 m) in 2012. The seedling endophyte status was checked by microscopy on leaf sheaths following the same protocol above mentioned, and plants from fungicide-treated and non-treated seeds were 0% and 100% infected with the endophyte respectively. Seeds produced from these two plant groups were labelled as EF and EI, respectively. Seed increases from this original source of EF and EI seeds are produced every year in our facilities, and the contrasting endophyte infection rate has been maintained across generations (0% and 100%, respectively).

A pot experiment was conducted in a greenhouse with constant temperature (temperature 26 ± 2 °C, humidity 42 ± 2%) for 3 months at the College of Pastoral Agricultural Science and Technology in 2020. Fresh seeds were obtained from the 2019 seed increase (recall that seed increases have been produced every year since the generation of EF and EI seeds in 2012). The soil substrate used in the experiment was a combination of peat soil and vermiculite (2:1 volume), sterilized at 180 °C for 2 h in an oven. A total of 200 plastic pots (upper diameter 12 cm, lower diameter 10 cm, height 13.5 cm, weight 30 g) lined with filter papers of 90 mm of diameter at the bottom were filled with 250 g of the sterilized soil substrate. Seeds of *A. inebrians* were sown in the 200 pots (100 pots for EI plants and 100 pots for EF plants), with three seeds per pot. The endophyte infection status was checked by microscopy in a single tiller per plant and confirmed that all EI plants were infected with the endophyte, while all EF were free of the endophyte. The pots were then watered daily according to the water-holding capacity (WHC) of each pot, maintaining 30% of soil moisture content as determined from the relative saturated moisture content (RSMC) at the start of the experiment. The RSMC represents the ratio of the actual soil moisture content to the potential soil moisture saturation, both determined by measuring the moisture contents in 10 pots [17]. In this experiment, 30% soil moisture was chosen as the control condition, since this moisture was the threshold that prevented plants entering in wilting point when watered at intervals of 24 h [19]. The positions of pots were changed every 3 days in the greenhouse. A month post sowing, most seedlings showed second true leaves, and a single plant per pot was retained based on similar size. The retained plants were watered with half-strength Hoagland nutrient solution once a week for 4 weeks.

After 2 months of growth, the watering of plants was ceased, and pots were weighed until the WHC of each pot reached a soil moisture content of 15% according to the RSMC. At this point, a total of 60 EI and 60 EF pots with plants of similar size were selected and pruned to a height of 10 cm from the soil substrate surface. Subsequently, three soil moisture conditions were established: 15% (low moisture), 30% (control moisture), and 60% (high moisture), each soil moisture condition with 20 EI and 20 EF plants. Plants were maintained at these soil moisture conditions for 4 weeks. During the trial, each pot was weighed every evening at 18:00 h. The moisture content of each pot was adjusted on a daily basis. After 4 weeks, 18 EI and 18 EF plants per soil moisture treatment were selected, and subsequently, six plants were randomly chosen and grouped together to generate single experimental replicates (three replicates per combination of treatments) for measurements. All leaf blades from each plant were harvested and pooled together within their respective plant group in aluminum foils, frozen in liquid nitrogen, and stored at – 80 °C until subsequent determination of plant oxidative damage, antioxidant contents, and gene expression levels.

#### Determination of the Oxidative Damage and Antioxidant Contents

Distinct kits of enzyme-linked immunosorbent assays (ELISA) (FANKEL Industrial Co. Ltd., Shanghai, China) were employed to determine the contents of MDA (an indicator of oxidative damage), enzymatic antioxidants (i.e., CAT, POD, SOD), and non-enzymatic antioxidants (i.e., AsA, Car, GSH, Pro) [39, 40]. Following kit instructions, measurements were performed as follows: 0.5 g of freeze-dried leaf blade samples was ground, and 5 mL of phosphate-buffered saline solution (PBS, pH 7.4) was added and centrifuged at 750 (×g) for 20 min at 4 ℃ using a refrigerated centrifuge (H-1850R, Xiangyi®, Hunan, China). Aliquots of 10 µL of supernatants were placed within wells of microtiter plates and diluted in 40 µL of PBS solution that included equal volumes of MDA and antioxidant standards. Plates were incubated for 30 min at 37 °C and washed five times with washing solution to remove unbound substances. The 50 µL of chromogen solution A and the same volume of chromogen solution B were added and incubated for 15 min at 37 ℃ in the dark. A volume of 50 µL of termination solution was added to the wells, and the optical density (OD) value of each sample was measured at 450 nm using a microplate reader (DR6000, Hach, Loveland, CO, USA). The limit of detection (LOD) and limit of quantification (LOQ) for the compounds were 0.10 and 12 μg∙g^−1^ for MDA, 0.30 and 9 U∙g^−1^ for CAT, 0.0006 and 0.0028 U∙g^−1^ for POD, 0.12 and 4.20 U∙g^−1^ for SOD, 0.10 and 80 μg∙g^−1^ for AsA, 1 and 200 μg∙g^−1^ for Car, 1 and 480 μg∙g^−1^ for GSH, and 0.001 and 0.02 μg∙g^−1^ for Pro. Separated calibration curves were performed for the distinct compounds that were measured and each curve was generated from specific standards. The contents of MDA and antioxidants were calculated by comparing the OD values with their corresponding calibration curves.

### RNA Extraction and Transcriptome Sequencing

RNA-seq was used to quantify the gene expression of plant transcriptomes. RNA was extracted from leaf blade samples (recall that there were three replicates per combination of treatments). The extraction was performed on 3 µg of ground leaf blades using the TRIzol reagent (Invitrogen, Carlsbad, CA, USA). The extracted RNA was cleaned with the RNeasy Plant Mini Kit (Qiagen, Valencia, CA, USA). The concentration and purity of the RNA were determined with a Qubit®2.0 fluorometer (Life Technologies, Inc., USA) and the RNA integrity number (RIN) with an Agilent 2100 Bioanalyzer (Agilent Technologies, Inc., USA). To construct the sequencing library, the NEBNext® Ultra™ RNA Library Prep Kit for Illumina® (NEB, San Diego, CA, USA) was utilized, and transcriptome sequencing was performed on the Illumina HiSeq-PE250 platform by Biomarker Technologies Company (Beijing, China). The sequencing depth allowed to capture plant RNAs.

For comparing the expression of plant genes, EF and 30% soil moisture samples were used as controls while EI, 15%, and 60% samples were taken as the experimental groups. We performed gene expression comparisons to test the single effects of the endophyte status (EI = EI-30% vs. EF-30%), low soil moisture (15% = EF-15% vs. EF-30%), and high soil moisture (60% = EF-60% vs. EF-30%) and combined effects of the endophyte & low soil moisture (EI & 15% = EI-15% vs. EF-30%) and endophyte & high soil moisture (EI & 60% = EI-60% vs. EF-30%). The gene expression comparisons in EI and EF plants at different soil moisture conditions are shown in Table S1. A total of 138.42-Gb clean reads were obtained from sequencing samples, with around 6.34-Gb clean reads per sample and above 89.19% of Q30 bases. There were 92,964 unigenes (N50 of 1677 base pair (bp)), with a mean length of 865.18 bp and 60,242 transcripts longer than 1000 bp (24.54%). Genes were annotated using the Kyoto Encyclopedia of Genes and Genomes (KEGG) with the KEGG Orthology-Based Annotation System (KOBAS) software [41]. Genes were also annotated using the non-redundant protein sequences (NR) from the National Center for Biotechnology Information (NCBI), Protein family (Pfam), Clusters of Orthologous Groups of proteins (KOG/COG/eggNOG), a manually annotated and reviewed protein sequence database (Swiss-Prot), KEGG, and Gene Ontology (GO) databases. The annotated unigenes were 42,618. The expression level of each annotated gene was normalized based on transcript lengths and library size. The normalized gene expression levels were then calculated as fragments per kilobase per million mapped fragments (FPKM).

We investigated the expression of genes associated with antioxidant pathways in EI and EF plants at different soil moisture conditions. Specifically, we calculated and identified the differentially expressed gene (DEG)–related comparisons regarding the EI, 15%, 60%, EI & 15%, and EI & 60% effects. Plant genes associated with antioxidant pathways were identified according to KEGG_annotation, NR_annotation, Pfam_annotation, Swissprot_annotation, and GO_annotation. Our analysis was focused on plant genes associated with the enzymatic antioxidants POD and non-enzymatic antioxidants AsA, Car, GSH, and Pro pathways, as delineated in the KEGG pathways in https://www.genome.jp/kegg/ and BMK KEGG pathway data (see genes in Table S2). The expression of genes related to MDA, CAT, and SOD pathways was not affected by the EI, 15%, 60%, EI & 15%, and EI & 60% effects (see Table S3); therefore, these genes were not included in further analyses. The RNA-seq data used in the present study were deposited in the Sequence Read Archive (SRA) of the NCBI database under accession number PRJNA748183 and previously used for other studies (i.e., [20, 21]).

### Statistical Analysis

The effects of the plant endophytic status and soil moisture treatments on the contents of oxidative damage indicator MDA; enzymatic antioxidants CAT, POD, and SOD; non-enzymatic antioxidants AsA, Car, GSH, and Pro were analyzed using two-way analysis of variance (two-way ANOVA) in the SPSS 22.0 program assuming normal distribution of errors (SPSS Inc., USA). The Duncan method was used for post hoc analyses to test the difference among means. All analyses met the ANOVA assumptions (residual independence, normality, and variance homogeneity). The results are presented as mean ± SEM (standard error of the mean) with a significance level of *P* < 0.05. Graphs were generated using the Origin 2018 software (OriginLab Corporation, USA). The DEGs were determined utilizing the exactTest function of edgeR package in the R software (version 3.5.0), with fold change (FC) > 1.5 and a false discovery rate (FDR) < 0.05 [42].

## Results

### Effects of Endophyte and Soil Moisture Treatments on Plant Oxidative Levels

**Fig. 1:**
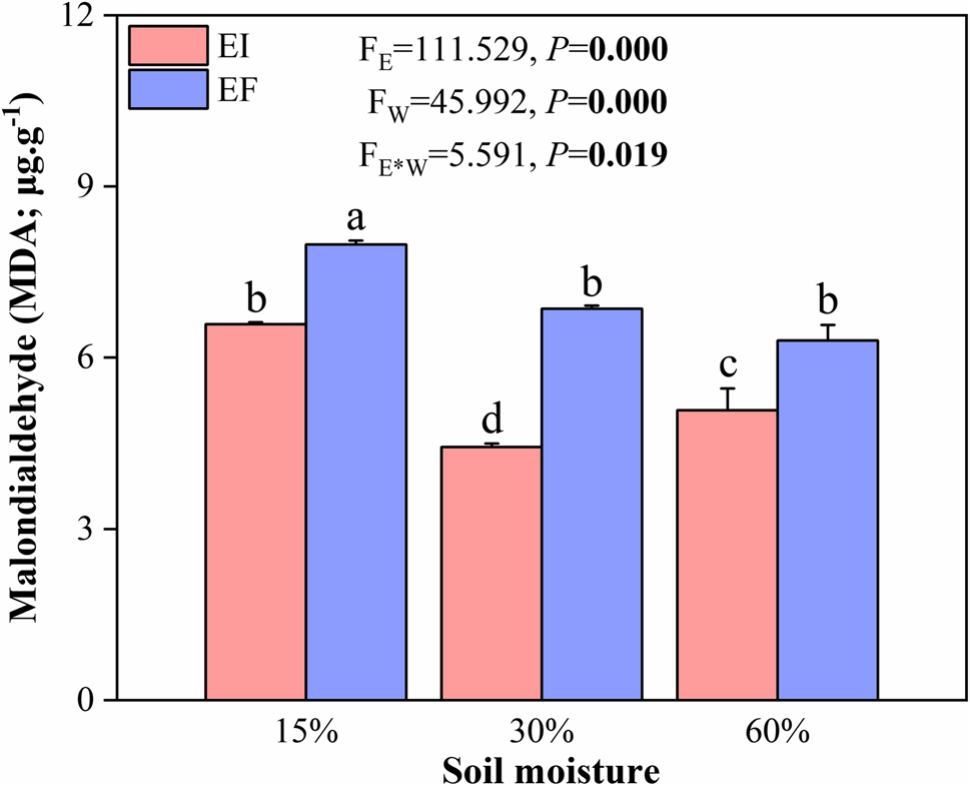
Malondialdehyde content in leaf blades of 3-month-old *Achnatherum inebrians* plants infected (EI) with or free (EF) of *Epichloë gansuensis* endophytes under 15%, 30%, and 60% soil moisture conditions. Each panel contains *F* and *P* values associated with the ANOVA factors plant endophyte status (*E*), soil moisture (*M*), and *E* × *M* interaction. Values in bold are significant at *P* < 0.05. Bar are means ± SEM (*n* = 3)

The effect of the endophyte on the oxidative damage indicator MDA depended on the soil moisture conditions (i.e., an endophyte × soil moisture interaction was observed) (Fig. 1).

At 30% soil moisture (control), the endophyte reduced MDA contents of plants. The MDA contents of plants were higher in EF-15% than EF-30%, but not different between EF-60% and EF-30%. The MDA contents did not differ between EI-15% and EF-30% but were lower in EI-60% than EF-30%. The endophyte reduced the MDA contents in 15% soil moisture conditions (Fig. 1).

### Effects of Endophyte and Soil Moisture Treatments on Plant Enzymatic Antioxidant Contents

**Fig. 2:**
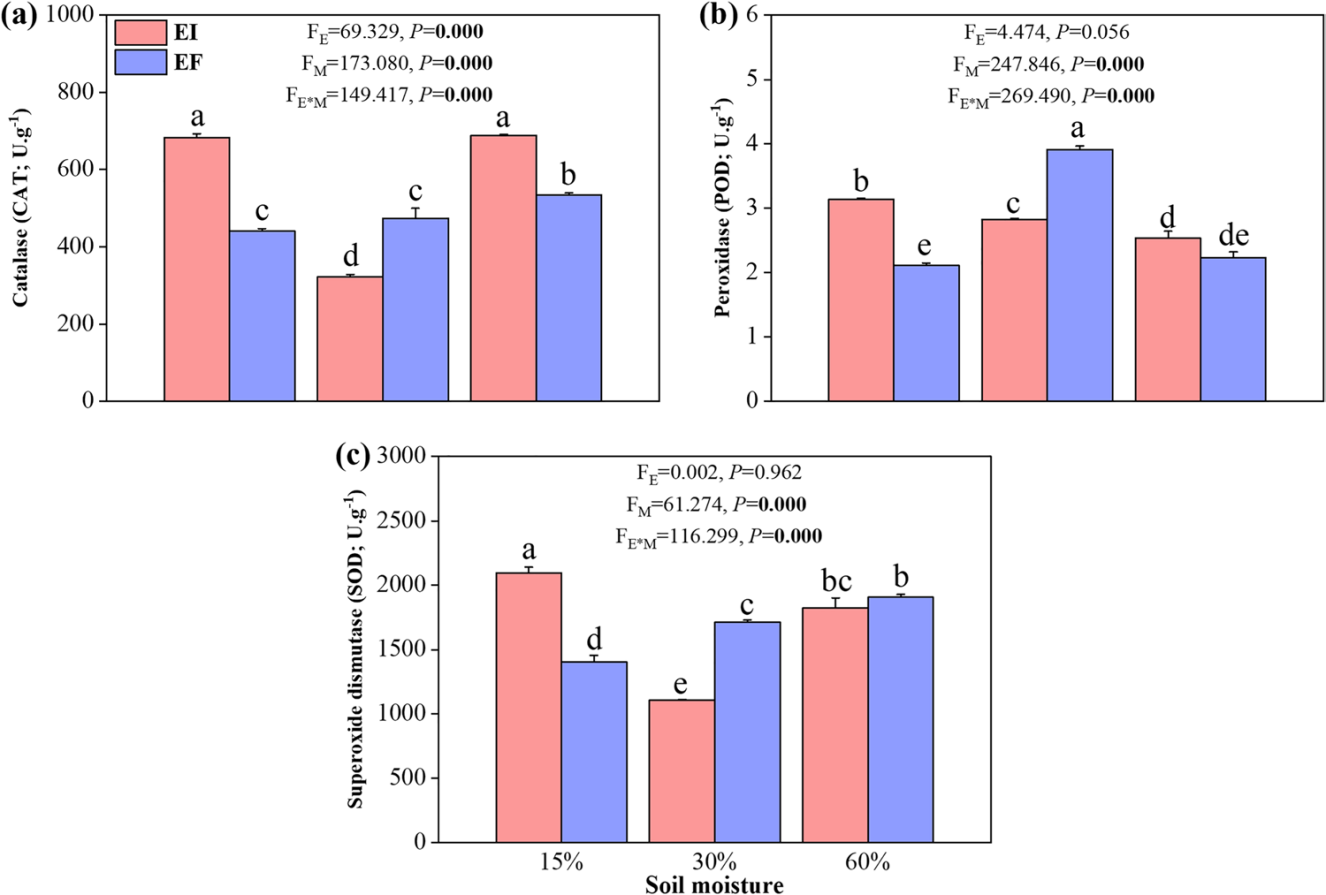
Contents of enzymatic antioxidants catalase (**a**), peroxidase (**b**), and superoxide dismutase (**c**) in leaf blades of 3-month-old *Achnatherum inebrians* plants infected with (EI) or free (EF) of *Epichloë gansuensis* endophyte under 15%, 30%, and 60% soil moisture conditions. Enzyme contents are expressed as units (U) per gram (g) of freeze-dried leaf samples. Each panel contains *F* and *P* values associated with the ANOVA factors plant endophyte status (*E*), soil moisture (*M*), and *E* × *M* interaction. Values in bold are significant at *P* < 0.05. Bar are means ± SEM (*n* = 3)

The effect of the endophyte on contents of CAT, POD, and SOD depended on the soil moisture conditions (i.e., an endophyte × soil moisture interaction was observed) (Fig. 2).

At 30% of soil moisture (control), the endophyte reduced the CAT contents. The CAT contents were not different between EF-15% and EF at 30%, but higher in EF-60% than EF-30%. The CAT contents were higher in both EI-15% and EI-60% than EF-30%. The endophyte increased CAT contents at 15% soil moisture (Fig. 2a).

At 30% of soil moisture (control), the endophyte reduced the POD contents. The POD contents were lower in both EF-15% and EF-60% than EF-30%. Similarly, the POD contents were also lower in EI-15% and EI-60% than EF-30%. The endophyte increased POD contents at 15% soil moisture (Fig. 2b).

At 30% soil moisture (control), the endophyte reduced the SOD contents. The SOD contents were lower in EF-15% than EF-30% but higher in EF-60% than EF-30%. The SOD contents were higher in EI-15% than EF-30% but not different between EI-60% and EF- 30%. The endophyte increased SOD contents in 15% soil moisture (Fig. 2c).

### Effects of Endophyte and Soil Moisture Treatments on Plant Non-enzymatic Antioxidant Contents

**Fig. 3:**
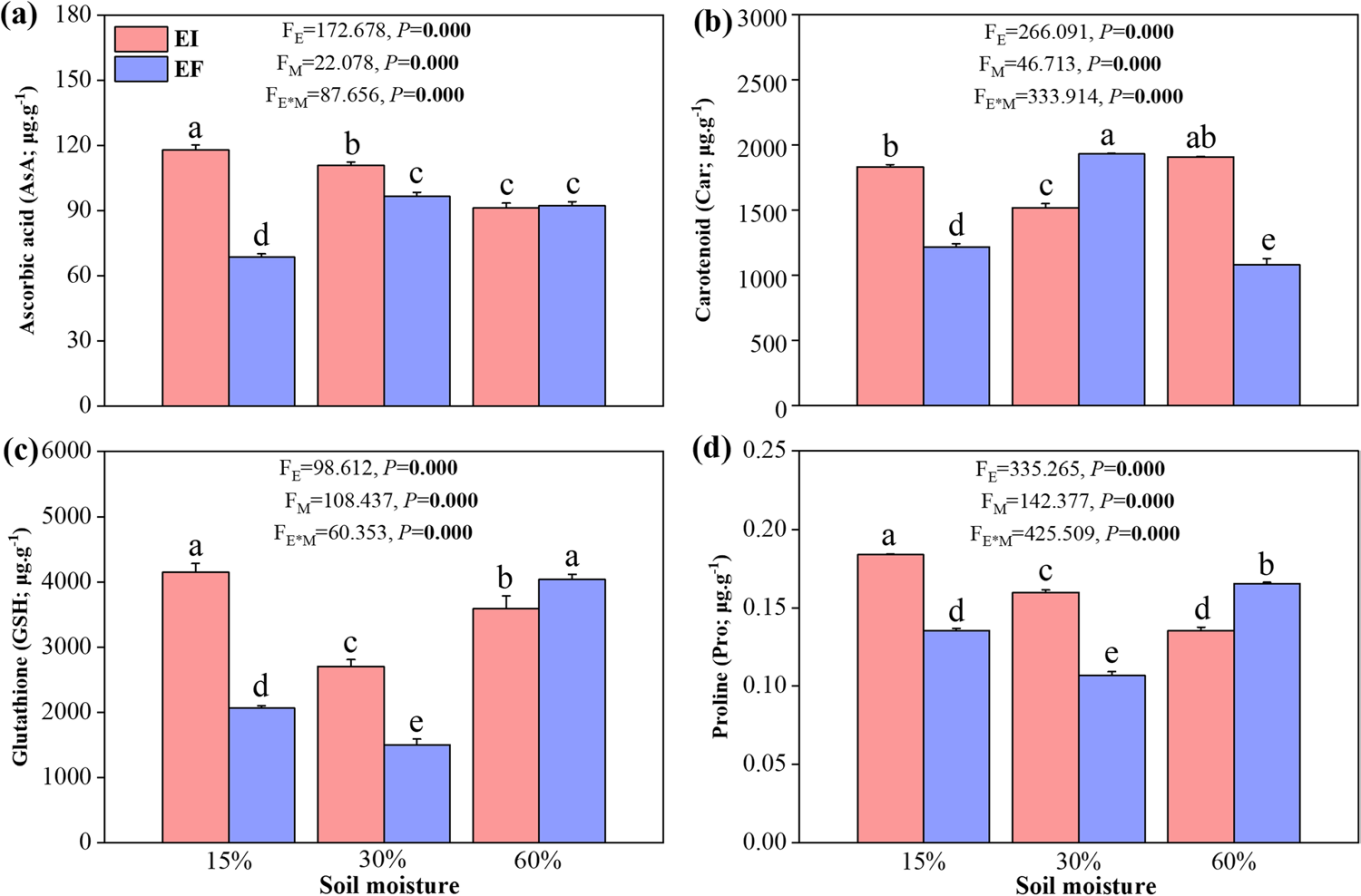
Contents of non-enzymatic antioxidants ascorbic acid (**a**), carotenoid (**b**), glutathione (**c**), and proline (**d**) in leaf blades of 3-month-old *Achnatherum inebrians* plants infected with (EI) or free (EF) of *Epichloë gansuensis* endophyte under 15%, 30%, and 60% soil moisture conditions. Contents of non-enzymatic antioxidants are expressed as micrograms per gram (g) of freeze-dried leaf samples. Each panel contains *F* and *P* values associated with the ANOVA factors plant endophyte status (*E*), soil moisture (*M*), and *E* × *M* interaction. Values in bold are significant at *P* < 0.05. Bar are means ± SEM *(n* = 3)

The effect of the endophyte on the contents of AsA, Car, GSH, and Pro depended on the soil moisture conditions (i.e., an endophyte × soil moisture interaction was observed) (Fig. 3).

At 30% soil moisture (control), the endophyte increased the AsA contents. The AsA contents were lower in EF-15% than EF-30% but not different between EF-60% and EF-30%. The AsA contents were higher in EI-15% than EF-30% but not different between EI-60% and EF-30%. The endophyte increased the AsA contents at 15% soil moisture (Fig. 3a).

At 30% soil moisture (control), the endophyte reduced the Car contents. The Car contents were lower in both EF-15% and EF-60% than EF-30%. The Car contents were lower in EI-15% than EF-30% but not different between EI-60% and EF-30%. The endophyte increased the Car contents at 15% soil moisture (Fig. 3b).

At 30% soil moisture (control), the endophyte increased the GSH contents. The GSH contents were higher at both EF-15% and EF-60% than EF-30%. Similarly, the GSH contents were also higher in EI-15% and EI-60% than EF-30%. The endophyte increased the GSH contents at 15% soil moisture (Fig. 3c).

At 30% soil moisture (control), the endophyte increased the Pro contents. The Pro contents were higher in both EF-15% and EF-60% than EF-30%. Similarly, the Pro contents were also higher in EI-15% and EI-60% than EF-30%. The endophyte increased the Pro contents at 15% soil moisture (Fig. 3d).

### Effects of Endophyte and Soil Moisture Treatments on Plant Genes Associated with Antioxidant Pathways

**Fig. 4:**
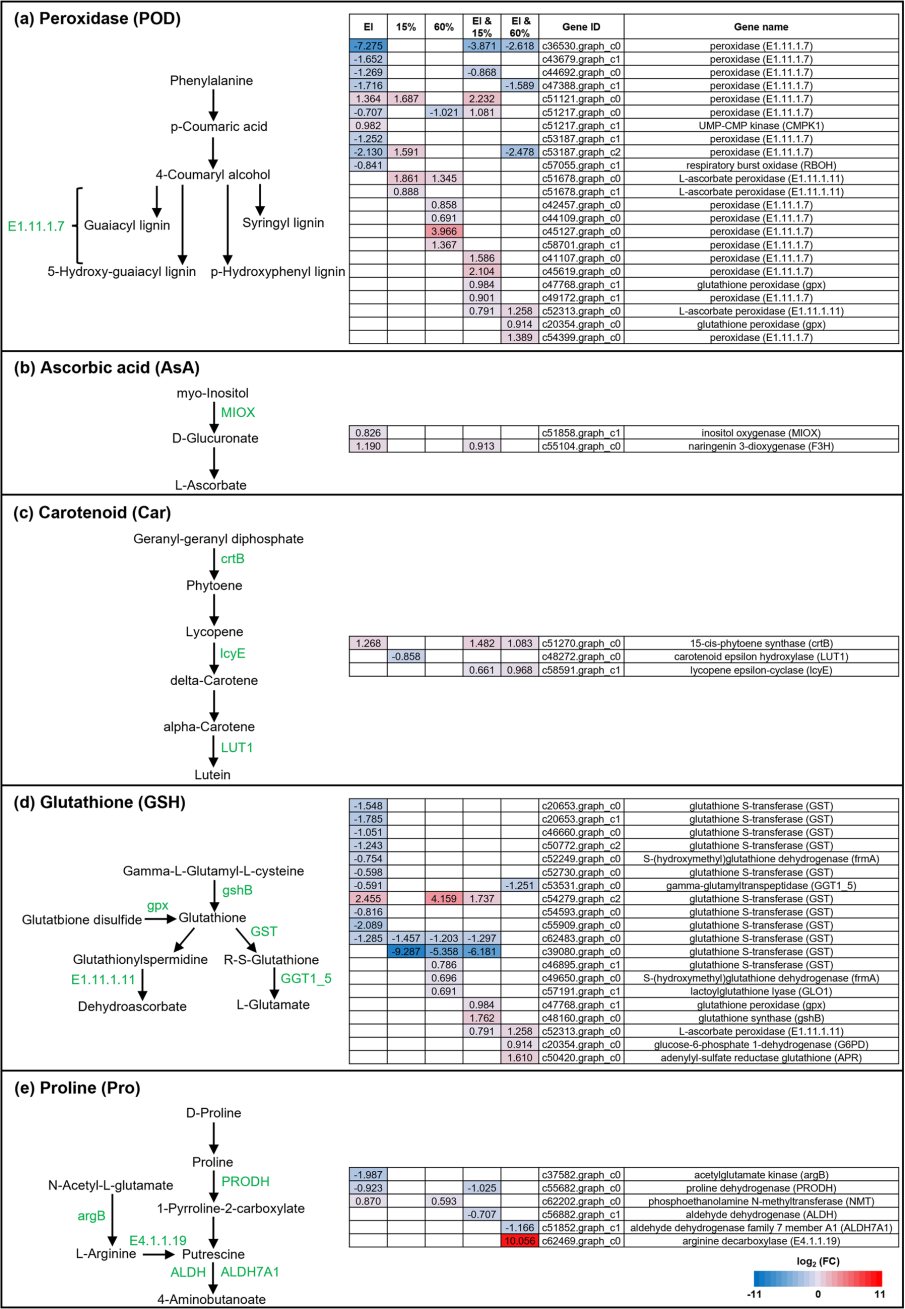
Changes in the expression levels of plant genes associated with antioxidant pathways associated with peroxidase (**a**), ascorbic acid (**b**), carotenoid (**c**), glutathione (**d**), and proline (**e**) in leaves of *Achnatherum inebrians* plants. The single effects of *Epichloë gansuensis *endophyte infection (EI), low soil moisture (15%), high soil moisture (60%), and the combined effects of *E. gansuensis* infection & low soil moisture (EI & 15%) and *E. gansuensis* infection & high soil moisture (EI & 60%) are shown. Simplified antioxidant pathways are depicted at the left, and differentially expressed genes (DEGs) are highlighted in green. Red and blue colors indicate significant differences in gene expression levels expressed as log_2_ fold change (FC) (*n* = 3)

We found 54 DEGs associated with antioxidant pathways in plant leaves, including 23 POD-, 2 AsA-, 3 Car-, 20 GSH-, and 6 Pro-related genes (Fig. 4, Table S2). These DEGs were primarily enriched in the pathways “Phenylpropanoid biosynthesis,” “Ascorbate and aldarate metabolism,” “Carotenoid biosynthesis,” “Glutathione metabolism,” and ”Arginine and proline metabolism” (Table S2).

In agreement with the endophyte-mediated reduction in POD contents in 30% soil moisture, the presence of endophyte reduced the expression of most of the plant POD genes detected (i.e., EI condition). In contrast with the soil moisture–based reduction in POD contents, the expressions of most plant POD genes were upregulated at 15% and 60% soil moisture conditions. In line with the endophyte/soil moisture–mediated reduction in POD contents, the plant POD genes that were downregulated in both EI & 15% and EI & 60% treatments showed higher changes in expression levels than the POD genes that were upregulated (Fig. 4a).

Consistent with the endophyte-mediated increase in AsA contents in 30% soil moisture, the endophyte increased the expression of plant *MIOX* and *F3H* genes that code for AsA biosynthetic enzymes (i.e., EI condition) [43]. Despite the reduction in AsA contents in 15% soil moisture, the expression of plant genes associated with AsA pathway were not different in this condition. In line with the unaltered AsA contents in high soil moisture, the expression of the plant AsA-derived genes did not change in the 60% condition. In line with the endophyte/soil moisture–mediated effects on AsA contents, the combined EI & 15% treatments increased the expression of the AsA biosynthesis gene *F3H* while the combined EI & 60% treatments did not alter the expression of plant AsA-derived genes (Fig. 4b).

In contrast with the endophyte-mediated reduction in Car contents in 30% soil moisture, the endophyte increased the expression of the plant *crtB* gene that codes for a Car biosynthesis enzyme. Similarly, against expectations based on the soil moisture–based reduction in Car contents, the expression of the Car-metabolizing plant *LUT1* gene was downregulated in the 15% condition while unchanged in the detected plant Car-derived genes in the 60% condition. Contrary to the endophyte/soil moisture–mediated effects on Car contents, both EI & 15% and EI & 60% treatments increased the expression of the Car biosynthesis plant genes *crtB* and *LcyE* (Fig. 4c).

Consistent with the endophyte-mediated increase in GSH contents in 30% soil moisture, the endophyte reduced the expression of most plant GST genes that code for enzymes that metabolize GSH (i.e., EI condition). In line with soil moisture–based increase in GSH contents, both 15% and 60% soil moisture conditions also reduced the expression of GSH-metabolizing plant GST genes. In line with the endophyte/soil moisture–mediated increase in GSH contents, the combined EI & 15% treatments simultaneously increased the expression of plant *gpx* and *gshB* genes encoding GST biosynthetic enzymes and reduced the expression of GSH-metabolizing GST genes. Similarly, the combined EI & 60% treatments reduced the expression of GSH-metabolizing GST genes (Fig. 4d).

In agreement with the endophyte-mediated increase in Pro contents in 30% soil moisture, the endophyte reduced the expression of the plant gene *PRODH* that codes for an enzyme that metabolizes Pro (i.e., EI condition). Paradoxically, considering the low soil moisture–based increase in Pro contents, the detected plant genes associated with Pro pathway were not differentially expressed in the 15% condition. In line with the high soil moisture–based increase in Pro contents, the plant *NMT* gene that codes for a Pro biosynthesis enzyme was upregulated in the 60% condition [44]. In line with the endophyte/soil moisture–mediated increase in Pro contents, EI & 15% and EI & 60% treatments reduced the expression of plant *PRODH*, *ALDH*, and *ALDH7A1* genes that code for enzymes that metabolize Pro (Fig. 4e).

## Discussion

We investigated the effects of the endophyte *E. gansuensis* and distinct soil moisture conditions on the level of oxidative damage, contents of antioxidants, and expression of plant genes related to antioxidant pathways in *A. inebrians* plants. As expected, the oxidative damage in EF plants, quantified via MDA contents, was elevated at low soil moisture but unaltered at high soil moisture. We hypothesized that the endophyte presence would reduce the plant oxidative damage specially at low soil moisture and our results agreed with this. The elevated oxidative damage in EF plants at low soil moisture was associated with a general reduction in antioxidant contents. We also expected that the endophyte would increase the antioxidant contents in plants specially at low soil moisture and the observed contents of both enzymatic and non-enzymatic antioxidants confirmed this hypothesis. The endophyte also increased the contents of most non-enzymatic antioxidants in plants grown at control soil moisture which may have contributed to the reduction in the oxidative damage. We predicted that the endophyte effects on the expression of plant genes associated with antioxidant pathways would be aligned with the changes in the patterns of accumulation/reduction of antioxidants across soil moisture conditions and as expected, the endophyte-mediated increases in antioxidant contents were generally associated with changes in the expression of plant genes that contributed to the accumulation of antioxidants in plant tissues.

Abiotic stresses generally trigger oxidative damage in plants [45]. Our results showed that the association of plants with *Epichloë* endophytes reduced the oxidative damage caused by the water restriction by 17.55%. *Epichloë*-mediated reductions in oxidative damage have also been documented in *Roegneria kamoji*, *Lolium perenne*, and *Festuca sinensis* plants subjected to water deficit [46–48]. In addition to drought, the presence of *Epichloë* endophytes in plants also alleviates the oxidative damage caused by other environmental stressors. For instance, the endophyte *E. gansuensis* reduced the MDA contents of plants grown in heavy metal–contaminated soils or exposed to fungal phytopathogens [31, 49]. Low MDA contents were also observed in *Hordeum brevisubulatum–Epichloë bromicola* plants grown in soils with elevated salinity [50].

*Epichloë* endophytes increased the contents of most antioxidants in plants grown under low soil moisture. Similar results have been documented in other plant-endophyte associations. *Epichloë* endophytes enhanced the contents of APX, CAT, and GR in *Festuca arizonica* and *Achnatherum robustum* plants subjected to drought stress [29]. The *Epichloë* presence in drought-treated *E. dahuricus* plants increased the contents of the antioxidants APX, CAT, POD, SOD, and Pro [36]. Antioxidants CAT, POD, and polyphenol oxidase (PPO) were also higher in endophyte-infected compared to endophyte-free *L. perenne* plants that were grown under water deficit [51]. In agreement with our hypothesis, the *Epichloë*-mediated enhancement in plant antioxidant contents at low soil moisture was correlated with reduced oxidative damage [27]. This pattern of change in antioxidant/oxidative damage has been observed in several plant-*Epichloë* associations (e.g., [52–54]). For instance, increased contents of APX, CAT, POD, SOD, GR, and Pro and reduced levels of ROS were documented in endophyte-symbiotic *E. dahuricus*, *F. arizonica*, and *A. robustum* plants grown at low soil moisture [29, 33]. It is expected that the combined effect of endophytes increasing antioxidant contents and reducing oxidative damage substantially enhance the fitness of their plant hosts especially under stress situations (e.g., [55, 56]). In fact, previous published findings have shown a higher performance of *A. inebrians* plants infected with *E. gansuensis* endophytes compared to endophyte-free plants when subjected to water deficit [17, 19, 20]. It is worth mentioning that *E. gansuensis* also increased the contents of many antioxidants of *A. inebriants* plants under the control soil moisture condition, and these enhanced antioxidant contents may have also contributed to alleviate the oxidative damage caused by the water deficit treatment [48, 57].

Although the endophyte-mediated enhancement in plant antioxidants has been extensively documented [58–60], the mechanisms by which endophytes alter antioxidant contents in plants are not entirely clear. Our results suggest that at least part of the *Epichloë*-mediated increase in antioxidant contents in plants was caused by the endophyte stimulation of the host production of antioxidants or the endophyte stabilization of host antioxidants. Regulation of the plant antioxidant production by endophytes has been previously reported. Increased GSH contents in cadmium-treated *Sedum alfredii* plants associated with *Sphingomonas* sp. strain SaMR12 were apparently caused by the positive effects of the bacterium on the upregulated expression of plant *GS* and *GSH1* genes that coded for antioxidant biosynthetic enzymes [61]. The arbuscular mycorrhizal fungus *Funneliformis mosseae* increased APX, CAT, and GR antioxidant contents in *Citrullus lanatus* plants grown in contrasting salinity-alkalinity conditions via upregulating the expression of plant genes associated with the biosynthesis of these antioxidants [36]. Similarly, the presence of the endophyte *Piriformospora indica* in *Citrus sinensis* plants increased the expression of plant genes *CsPOD* and *CsCAT1* that coded for biosynthetic enzymes of CAT and POD antioxidants [62]. It is worth mentioning that although most of the plant gene expression results were aligned with the reported antioxidant contents, there was a lack of alignment in the findings associated with the Car. This may be because the biosynthesis of Car is complex, involving multiple enzymes and reaction steps, therefore it is likely that the expressions of the few genes reported in the present study were not sufficient to influence the total content of the antioxidant [63, 64].

## Conclusion

Our findings provide strong circumstantial evidence that the drought-derived oxidative damage in *A. inebrians* plants was probably alleviated by ability of *Epichloë* endophytes to increase antioxidant contents in plants and upregulate plant genes related to antioxidant pathways. The endophyte-based alleviation of the plant oxidative damage may be associated with the endophyte’s capacity to provide antioxidants, promotes the host production of antioxidants, or stimulates the activities of the existing host antioxidants [7, 31, 57, 65, 66]. Plant gene expression results suggest that at least part of the endophyte-based reduction in oxidative damage would be associated with the fungal regulation of plant antioxidants. Furthermore, studies would be warranted to determine the relative contribution of the *Epichloë*-derived antioxidants in the alleviation of the plant oxidative damage and if endophytes can alter kinetic of reaction of plant antioxidants. Our findings provide indicative evidence regarding the endophytic regulation of antioxidants in plants and suggest novel avenues for future research to understand how plant-endophyte associations respond to environmental stresses.

### Supplementary Information

Below is the link to the electronic supplementary material.

Supplementary file1 Table S1: Gene expression comparisons in leaf blades of three-month-old *Achnatherum inebrians* plants infected (EI) with or free (EF) of *Epichloë gansuensis* endophytes under 15%, 30%, and 60% soil moisture conditions. Table S2:Genes associated with peroxidase, ascorbic acid, carotenoid, glutathione, and proline pathways based on KEGG in leaf blades of three-month-old *Achnatherum inebrians* plants infected (EI) with or free (EF) of *Epichloë gansuensis* endophytes under 15%, 30%, and 60% soil moisture conditions. Table S3:Genes associated with malondialdehyde, catalase, and superoxide dismutase in leaf blades of three-month-old *Achnatherum inebrians* plants infected (EI) with or free (EF) of *Epichloë gansuensis* endophytes under 15%, 30%, and 60% soil moisture conditions. (XLSX 11809 KB)

## Data Availability

The biochemical data measured in the current study are available from the corresponding author on reasonable request. The RNA-seq data is deposited in the Sequence Read Archive (SRA) of the NCBI database under accession number PRJNA748183.
